# Evaluation of methods to classify ipsilateral breast tumour recurrences as local recurrence or new primary tumour

**DOI:** 10.1038/s41523-025-00850-8

**Published:** 2026-02-13

**Authors:** S. Blacker, JM. Boyle, L. Wang, DR. Withrow, C. Delon, D. Dodwell, M. Verrill, A. Lemanska, SE. Pinder, AE. Frampton, K. Horgan, DA. Cromwell

**Affiliations:** 1 Clinical Effectiveness Unit, The Royal College of Surgeons of England, London, UK; 2 Faculty of Health and Medical Sciences, University of Surrey, Guildford, UK; 3 Department of Health Services Research & Policy, London School of Hygiene & Tropical Medicine, London, UK; 4 Nuffield Department of Population Health, University of Oxford, Oxford, UK; 5 Northern Centre for Cancer Care, Freeman Hospital, Newcastle-upon-Tyne, UK; 6 Imperial Clinical Trials Unit, School of Public Health, Imperial College London, London, UK; 7 School of Cancer & Pharmaceutical Sciences, King’s College London, London, UK; 8 Department of Breast Surgery, St James’s University Hospital, Leeds, UK

**Keywords:** Cancer, Oncology

## Abstract

Ipsilateral breast tumour recurrence (IBTR) may represent a true local recurrence (LR) from residual malignancy or a new primary (NP) tumour, with important implications for prognosis and treatment. However, no classification system exists to distinguish between these entities. This systematic review of studies evaluating classification methods for IBTR as LR or NP, identified 19 studies reporting 25 systems. Most were clinicopathological (21/25) and four were genomic. Tumour location (72%) and histological subtype (68%) were the most frequently applied criteria. IBTR rates ranged from 2 to 12%, with NP proportions between 13–82% and LR between 18–87%. Time to recurrence was shorter for LR than NP. Across studies, NP was consistently associated with superior survival outcomes. The methodological quality of included studies constrains the certainty of findings. Validation of clinicopathological and genomic criteria is needed before a classification system can be recommended, but pragmatic clinicopathological decisions remain essential in the interim.

## Introduction

Ipsilateral breast tumour recurrence (IBTR) following breast conserving surgery (BCS) for early invasive breast cancer (EIBC) occurs in 1.9% to 11.1% of cases^1^. Patients who experience recurrence have an increased risk of distant metastases and higher breast cancer-related mortality, with a 5-year survival rate of ~80%^2–5^. However, it is thought that outcomes for IBTR are influenced by whether the ‘recurrence’ represents a true local recurrence (LR), arising from growth of residual malignancy (in situ or invasive) or a new primary (NP) tumour, arising in the residual breast tissue. Studies report that disease free and overall survival are superior in patients with NP tumours, whereas LR tumours are associated with a higher incidence of metastatic disease^6–8^. The current a lack of established criteria to help clinicians differentiate between LR and NP tumours, presents two key challenges in the management of IBTR.

First, the inability to distinguish LR from NP makes tailored management and prognostication challenging. Although mastectomy remains the traditional standard of care for IBTR, a national survey of NHS services in the UK reported considerable variation in practice between breast units, reflecting the absence of evidence or UK-specific guidelines to inform management^9^. There is evidence that further BCS, with or without re-irradiation, is increasingly offered as an alternative to mastectomy in selected cases^2,10^. Internationally, the 2020 American National Comprehensive Cancer Network guidelines recommend mastectomy for isolated IBTR, with radiotherapy only if not previously administered, but do not propose distinct strategies for NP and LR, reflecting the current inability to reliably make this distinction^11^.

Second, the absence of consistent classification criteria limits the quality of data that can be captured in national registries, and, by extension, the strength of the evidence base that informs practice. Breast multidisciplinary teams (MDT) in England are expected to make the distinction between LR and NP when recording second ipsilateral breast tumours in submissions to the Cancer Outcomes and Services Data set (COSD)^12^. Yet, without a widely accepted and reliable method for making this distinction, variation in reporting is inevitable. Establishing consistent criteria would improve the quality of national datasets, enable more meaningful outcome monitoring and ultimately support the development of evidence-based guidance for IBTR management.

Multiple methods have been proposed to assist clinicians in distinguishing between NP and LR breast tumours. In the absence of an accepted standard for making this distinction, the concept of construct validity, which describes the extent to which a classification system accurately reflects the theoretical concept it is intended to measure, is pertinent for evaluating these approaches^13,14^. For IBTR, a proposed classification system could be considered to demonstrate construct validity if tumours classified as NP and LR consistently exhibit distinct characteristics and clinical outcomes. This review critically examines the evidence, construct validity, strengths and limitations of reported methods used to classify IBTR as either NP or LR tumours.

## Results

**Fig. 1:**
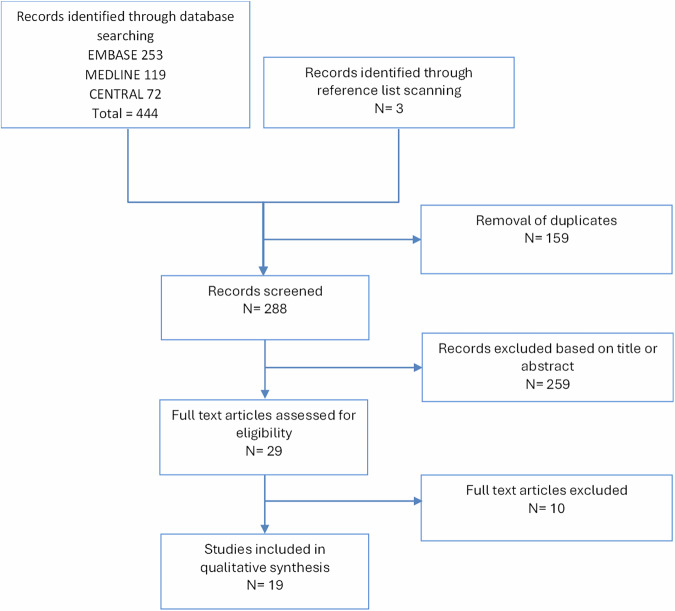
Flow chart of search strategy and study selection process.

The electronic database search identified 444 records, which were reduced to 285 after removal of duplicates. An additional 3 studies were identified through reference list screening, resulting in 288 studies for title and abstract screening. Of these, 259 were excluded based on the predefined criteria. The remaining 29 articles were assessed in full, with 19 meeting the eligibility criteria for inclusion^6–8,15–30^. The study selection process is summarised in Fig. 1.

### Study characteristics

Table 1 summarises the characteristics of the 19 included studies, all of which employed a cohort design and applied their classification system(s) retrospectively. Thirteen studies (68%) were conducted within a single centre^6,15,17–23,26–28,30^.

**Table 1:** Key characteristics of studies selected for the systematic review (*n* = 19)

Author (year)	Country, setting	Recruitment period	Inclusion criteria	Total patient number	Method assessing classification construct validity	Outcome after IBTR
*Studies of CP classification systems*						
Gujral et al.^16^	UK, MC	1986-1998	BCS for T1-3 N0-1 M0 invasive BC with clear margins <75 years.	1410	TTR, CP characteristics	NR
Huang et al.^17^	USA, SC	1970-1994	BCS for BC.	1339	TTR, CP characteristics, CBC rate	OS, DSS, DMFS
Jobsen et al.^18^	The Netherlands, SC	1985-2015	BCS for IBC.	4359	TTR, CP characteristics	DSS, DMFS
Komoike et al.^8^	Japan, MC	1986-1993	BCS & ALND for unilateral BC < 3 cm.	1901	TTR, CP characteristics	OS, DFS, DMFS^a^
Krauss et al.^19^	USA, SC	1980-1997	BCS for stage 1-2 IBC.	1448	TTR, Concordant histology	OS, DSS
Laird et al.^20^	USA, SC	1998-2008	BCS for stage 1-3 IBC.	3932	TTR, Concordant histology	OS, DFS, DMFS^b^
Nishimura et al.^23^	Japan, SC	1986-2001	BCS for stage 1-2 IBC.	2137	TTR, CP characteristics, CBC rate	OS, DMFS
Panet-Raymond et al.^24^	Canada, MC	1989-1999	BCS for T1-2 N0-1 M0 IBC with clear margins.	6020	TTR, CP characteristics, CBC rate	OS, DSS, DMFS
Sakai et al.^26^	Japan, SC	1986-2007	BCS for BC, IBTR surgically treated 1998-2007.	3876	TTR, CP characteristics	DMFS
Sarsenov et al.^27^	Turkey, SC	1998-2007	BCS for EIBC (T1-2 N0-1 M0).	1400	TTR, CP characteristics	OS
Smith et al. ^28^	USA, SC	1970-1990	BCS for stage 0-2 BC.	1152	TTR, CP characteristics	OS, DSS, DMFS
Wang et al. ^7^	USA, MC	1990-2005	BCS for stage 1-3 IBC.	168,427	TTR, CP characteristics	DSS
West et al. ^29^	Canada, MC	NR	BCS for T1-2 N0-1 M0 IBC.	NR	TTR, CP characteristics, TIL biomarkers	NR
Yi et al. ^6^	USA, SC	1970-2005	BCS for BC.	5660	TTR, CP characteristics, CBC rate	OS, DFS
Yoshida et al.^30^	Japan, SC	1987-2005	BCS for IBC.	2075	TTR, CP characteristics	OS
*Studies of genomic classification systems*						
Fernandez-Abad et al.^15^	Spain, SC	NR	BCS or mastectomy for BC.	33	TTR, Concordance to CP classification	NR
McGrath et al.^21^	USA, SC	1980-2004	BCS for stage 1-2 IBC.	NR	TTR, Concordance to CP classification	OS, DSS, DFS
Nakagomi et al.^22^	Japan, SC	1999-2018	BCS for BC.	1881	TTR, CP characteristics	NR
Rassy et al.^25^	France & Italy, MC	1992-2018	BCS or mastectomy for ER positive IBC.	NR	TTR, CP characteristics	OS, DFS

*BC* breast cancer, *BCS* breast conserving surgery, *CBC* contralateral breast cancer, *CP* clinicopathological, *DFS* disease free survival, *DMFS* distant metastasis free survival, *DSS* disease specific survival, *ER* oestrogen receptor, *IBC* invasive breast cancer, *IBTR* ipsilateral breast tumour recurrence, *MC* multicentre, *NR* not reported, *OS* overall survival, *SC* single centre, *TIL* tumour infiltrating lymphocytes, *TTR* time to recurrence, *UK* United Kingdom, *USA* United States of America.

^a^Local relapse free survival.

^b^Second locoregional recurrence free survival.

Most studies (17; 90%) included only patients treated with BCS for their PBC. In the remaining studies, the study population comprised patients treated with either mastectomy or BCS as their initial surgical treatment^21,25^. There was considerable variability in study eligibility criteria regarding disease stage. Only six studies (32%) included patients whose PBC was non-invasive^6,15,17,22,26,28^, one study (5%) did not report invasive status^8^ and the remaining 12 (63%) included those with invasive disease only.

IBTR was most commonly defined as the first recurrence event occurring in the ipsilateral breast (12; 63%)^6,8,16–20,24,26–30^. Few manuscripts clarified whether the recurrence was invasive or non-invasive. Many studies subsequently excluded patients who experienced regional or distant recurrence prior to, or concurrently with their IBTR, although the specific exclusion criteria varied between studies.

Radiotherapy receipt for the PBC also varied amongst study participants. In nine of the 19 studies (47%), all participants received either whole or partial breast radiotherapy^7,16–21,27,28^. Four studies (32%) did not report details regarding radiotherapy use^6,8,15,26^, while in the remaining six studies, receipt of radiotherapy by study participants ranged from 42 to 98%^22–25,29,30^. Details of study radiotherapy regimes are supplied in Supplementary 1, alongside additional study design features, including exclusion criteria and follow up periods from PBC and IBTR.

Most studies (15; 79%) evaluated one or more classification systems based on clinicopathological features^6–8,16–20,23,24,26–30^, while the remaining four studies (21%) focused on genomic comparisons^15,21,22,25^. While the majority of studies report a single classification system, two (Jobsen et al.^18^ and Yi et al.^6^) reported multiple systems—six and two, respectively. Jobsen et al. re-evaluated four existing systems whose original studies are independently included in this review and additionally proposed two novel classification systems^18^.

### Assessment of construct validity

None of the studies explicitly stated an intention to establish the construct validity of their proposed classification systems for distinguishing NP and LR breast tumours. Time to IBTR was the most commonly used characteristic that was expected to differ between NP and LR tumours, and was reported in some form across all included studies. All studies included comparative analyses of various tumour characteristics between the NP and LR groups to explore whether these were distinguishing features. Among the studies assessing genomic classification methods, two out of four (50%) compared their proposed systems to existing clinicopathological classification systems to support claims of superior validity^15,21^. Other characteristics used to assess construct validity included West et al.^29^, who used differences in tumour-infiltrating lymphocyte (TIL) presence to validate their clinicopathological classification system, whilst rates of contralateral breast carcinoma (CBC) were compared between NP and LR groups in four studies^6,17,23,24^.

### Variation in definitions and distribution of NP and LR tumours

**Fig. 2:**
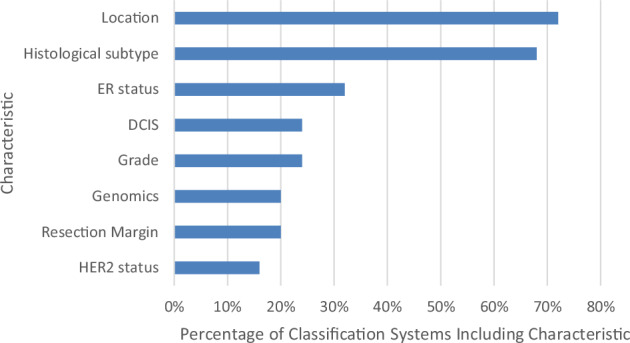
Summary of characteristics used to distinguish new primary tumours and true local recurrences. Abbreviations: DCIS ductal carcinoma in situ, ER oestrogen receptor, HER2 human epidermal growth factor receptor 2.

Tables 2 and 3 summarise the 25 classification systems and definitions of IBTR, NP and LR used across the 19 studies in this review. Figure 2 summarises the characteristics used to distinguish NP and LR tumours. Among the 25 classification systems reviewed, tumour location and histological subtype (e.g. ductal, no special type, lobular, etc.) were the most commonly used clinicopathological features to differentiate between NP and LR tumours, applied in 18 (72%) and 17 (68%) systems, respectively. Other features included histological grade (24%), oestrogen receptor (ER) status (32%), human epidermal growth factor receptor 2 (HER2) status (16%), resection margin status (20%), presence of ductal carcinoma in-situ (DCIS) (24%), and genomic clonality (20%). Of the five classification systems incorporating genomic clonality, four relied exclusively on genomic data^15,21,22,25^, while one combined genomic information with tumour location and histology ^28^.

**Table 2:** A comparison of clinicopathological classification systems

Author	IBTR criteria	IBTR exclusion	Method Description	Localisation	Histological subtype	Grade	ER status	HER2 status	Resection Margin	Presence of DCIS	Genomics
Gujral et al.^16^	First recurrence event in ipsilateral breast	NR	LR = concordant location (quadrant), histology & grade (or higher). NP = distinct location, histology & lower grade. Additional ‘Likely LR & NP’ categories where above criteria inconclusive.	X	X	X					
Huang et al.^17^	First recurrence event in ipsilateral breast	Poor data quality in records	LR = concordant location (within 3 cm) and histology/progression from DCIS to invasive disease. NP = failed to meet above criteria/change from invasive to DCIS.	X	X					X	
Jobsen et al.^18^ - Huang	First recurrence event in ipsilateral breast	IBTR after or at same time as regional recurrence or DM	LR = concordant location (within 3 cm) and histology.	X	X						
Jobsen et al.^18^ - Komoike	First recurrence event in ipsilateral breast	IBTR after or at same time as regional recurrence or DM	LR = concordant location and histology or positive margins (PBC). NP = distinct location and positive/negative margins (PBC) or concordant location with negative margins and distinct histology.	X	X				X		
Jobsen et al.^18^ - Morphology	First recurrence event in ipsilateral breast	IBTR after or at same time as regional recurrence or DM	LR = concordant histology, grade, ER & HER2 status (only change of grade 3–1 deemed significant).		X	X	X	X			
Jobsen et al.^18^ - Panet-Raymond	First recurrence event in ipsilateral breast	IBTR after or at same time as regional recurrence or DM	NP = If meets any of the following criteria: 1. change of histology; 2. grade 3 to 1; 3. ER negative to positive; 4. location > 3 cm or distant.	X	X	X	X				
Jobsen et al.^18^ - Twente	First recurrence event in ipsilateral breast	IBTR after or at same time as regional recurrence or DM	LR = concordant location (boost area/adjacent), histology, grade, ER & HER2 status (only change of grade 3–1 deemed significant).	X	X	X	X	X			
Jobsen et al. ^18^ - Yi	First recurrence event in ipsilateral breast	IBTR after or at same time as regional recurrence or DM	LR = concordant location (boost area), histology, ER & HER2 status.	X	X		X	X			
Komoike et al.^8^	First recurrence event in ipsilateral breast	IBTR after DM	LR = concordant location and histology or positive margins (PBC). NP = distinct location and positive/negative margins (PBC) or concordant location with negative margins and distinct histology.	X	X				X		
Krauss et al.^19^	First recurrence event in ipsilateral breast	IBTR after or at same time as DM. Recurrence location NR or lymphatic only.	LR = Concordant location (quadrant).	X							
Laird et al.^20^	First recurrence event in ipsilateral breast	IBTR after or at same time as DM. FU < 2 months from IBTR, unavailable pathology, or no breast surgery for IBTR.	LR = invasive tumour only. NP = in-situ component within or adjacent to invasive tumour.							X	
Nishimura et al.^23^	NR	NR	NP = negative margins (PBC) and IBTR contains in-situ component.						X	X	
Panet-Raymond et al.^24^	First recurrence event in ipsilateral breast	IBTR after regional recurrence or DM	NP = If meets any of the following criteria: 1. change of histology; 2. grade 3–1; 3. ER negative to positive; 4. location > 3 cm.	X	X	X	X				
Sakai et al.^26^	First recurrence event in ipsilateral breast	NR	LR = concordant location, positive margin +/- IBTR contains in-situ component. NP = distinct location or negative margins (PBC) and IBTR contains in-situ component.	X					X	X	
Sarsenov et al.^27^	NR	NR	LR = concordant location (quadrant) and histology. NP = non-concordant location (quadrant) and histology.	X	X						
Smith et al.^28^	First recurrence event in ipsilateral breast	NR	LR = concordant location, histology & flow cytometry. NP = Any of distinct location, histology, flow cytometry.	X	X					X	X
Wang et al.^7^	Invasive SBC	No surgery for IBTR, IBTR at same time as DM	LR = concordant location (quadrant) and histology, Concordant ER/PR status considered if tumour location unclear. NP = distinct location or histology.	X	X		X				
West et al.^29^	First recurrence event in ipsilateral breast where PBC & IBTR specimens available.	IBTR at same time as DM	NP = If meets any of the following criteria: 1. change of histology; 2. grade 3–1; 3. ER negative to positive; 4. location > 3 cm.	X	X	X	X				
Yi et al. - Method 2^6^	First recurrence event in ipsilateral breast	Poor data quality in records	LR = concordant location (within 3 cm) and histology.	X	X					X	
Yi et al - Method 2^6^	First recurrence event in ipsilateral breast	Poor data quality in records	LR = concordant location (within 3 cm), histology and ER/ HER2 status.	X	X		X	X			
Yoshida et al.^30^	First recurrence event in ipsilateral breast	NR	LR = concordant location or positive margins. NP = distinct location and positive or negative margins, concordant location with negative margins and distinct histology.	X	X				X		

*DCIS* ductal carcinoma in situ, *DM* distant metastases, *ER* oestrogen receptor, *FU* follow up, *HER2* human epidermal growth factor receptor 2, *IBTR* ipsilateral breast tumour recurrence, *LR* true local recurrence, *NP* new primary, *NR* not reported, PBC primary breast cancer, *SBC* second breast cancer.

**Table 3:** A comparison of genomic classification systems

*Studies of genomic classification systems*			
Author	**IBTR criteria**	**IBTR exclusion**	**Method Description**
Fernández-Abad et al.^15^	NR	NR	LR = PBC and IBTR shared at least one pathogenic mutation or amplification in at least one known driver gene related with breast carcinogenesis with primary tumour. NP = absence of shared mutations between PBC and IBTR.
McGrath et al.^21^	NR	NR	LR = Clonally related. NP = absence of shared mutations between PBC and IBTR.
Nakagomi et al.^22^	NR	NR	LR = PBC and IBTR shared at least one mutation, especially driver oncogenic mutations. NP = absence of shared mutations between PBC and IBTR.
Rassy et al.^25^	Any ipsilateral SBC with locoregional involvement and specimens available for PBC & IBTR.	Poor specimen quality	LR = Clonally related. NP = absence of shared mutations between PBC and IBTR.

*IBTR* ipsilateral breast tumour recurrence, *LR* true local recurrence, *NP* new primary, *NR* not reported, *PBC* primary breast cancer, SBC second breast cancer.

**Fig. 3:**
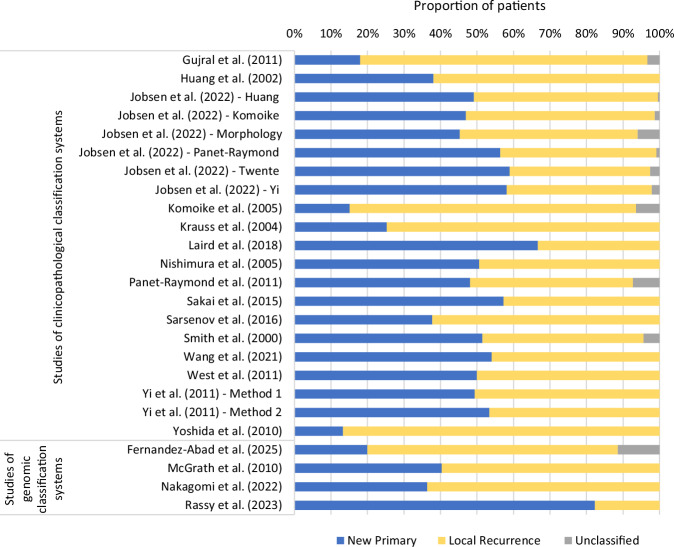
Distribution of new primary and true local recurrence tumours across the 25 classification systems.

Figure 3 summarises the distribution of NP and LR tumours across each classification system; additional details are provided in Supplementary 2. Among the 15 (79%) studies reporting both the total number of patients and the number of IBTR events, the IBTR rate ranged from 2 to 12%. The median follow up duration from the time of PBC diagnosis ranged from 5.4 to 13.0 years (IQR 6.1–11.1 years) across the nine studies providing this information. Among the seven studies reporting median follow up from the time of IBTR, this ranged from 2.1 to 7.0 years (IQR 4.8–5.9 years).

The proportion of patients labelled as having NP tumours across all classification systems ranged from 13% to 82%, while the proportion of patients labelled with LR tumours ranged from 18 to 87%. This substantial variation persisted within both clinicopathological (NP: 13–67%; LR: 33–87%) and genomic classification systems (NP: 20–82%; LR: 18–69%).

Ten classification systems included an ‘unclassified’ category, with 1–11% of tumours assigned to this group. Supplementary 3 provides a detailed summary of how key patient and tumour characteristics differed between NP and LR tumours across studies.

### Survival

Survival outcomes following IBTR were reported in 15 studies (79%)^6–8,17–21,23–26,28,30^. The survival outcomes reported varied across studies and included overall survival (OS), disease specific survival (DSS), distant metastasis free survival (DMFS), disease free survival (DFS) and second locoregional recurrence free survival.

Table 4 compares time to IBTR and survival outcomes for NP and LR tumours across the included studies. Across all classification systems, tumours classified as LR demonstrated a consistently shorter time to IBTR compared to NP tumours (IQR: LR 3.6–8.7 years vs NP 5.7–11.3 years). This difference was typically around 2 years within the clinicopathological classification systems LR but was more pronounced for several of the genomic classification systems.

**Table 4:** Time to IBTR and survival for NP and LR tumours for each classification system

Author	Mean time to IBTR (years)		Overall survival from IBTR diagnosis (Time horizon, %)		Disease Specific Survival from IBTR diagnosis (Time horizon, %)	
	NP	LR	NP	LR	NP	LR
Studies of clinicopathological classification systems						
Gujral et al.^16^	MD 6.2	MD 3	NR	NR	NR	NR
Huang et al.^17^	7.3	5.6	5 yr; 88	5 yr; 55	10 yr; 83	10 yr; 49
Jobsen et al.^18^ - Huang	13.0	10.1	NR	NR	^b^10 yr; 73	^b^10 yr; 67
Jobsen et al.^18^ - Komoike	13.0	10.0	NR	NR	^b^10 yr; 71	^b^10 yr; 68
Jobsen et al.^18^ - Morphology	12.9	9.8	NR	NR	^b^10 yr; 75	^b^10 yr; 65
Jobsen et al.^18^ - Panet-Raymond	12.7	10.0	NR	NR	^b^10 yr; 72	^b^10 yr; 67
Jobsen et al.^18^ - Twente	12.5	10.2	NR	NR	^b^10 yr; 75	^b^10 yr; 63
Jobsen et al.^18^ - Yi	12.2	10.3	NR	NR	^b^10 yr; 75	^b^10 yr; 64
Komoike et al.^8^	5.2	3.9	^a^10 yr; 92	^a^10 yr; 71.2	NR	NR
Krauss et al.^19^	MD 7.4	MD 5.7	8 yr; 73	8 yr; 79	NR	NR
Laird et al.^20^	MD 4.4	MD 4.9	5 yr; 92	5 yr; 60	NR	NR
Nishimura et al.^23^	4.6	3.1	5 yr; 91	5 yr; 76	NR	NR
Panet-Raymond et al.^24^	6.3	4.8	10 yr; 55	10 yr; 44	10 yr; 61	10 yr; 56
Sakai et al.^26^	4.8	2.6	NR	NR	NR	NR
Sarsenov et al.^27^	4.0	3.1	^b^5 yr; 95	^b^5 yr; 75	NR	NR
Smith et al.^28^	7.3	3.7	10 yr; 75	10 yr; 55	^b^10 yr; 90	^b^10 yr; 55
Wang et al.^7^	NR	NR	NR	NR	NR	NR
West et al.^29^	MD 7.7	MD 4.1	NR	NR	NR	NR
Yi et al. - Method 1^6^	7.6	5.4	NR	NR	NR	NR
Yi et al - Method 2^6^	7.4	5.5	NR	NR	NR	NR
Yoshida et al.^30^	MD 4.8	MD 2.6	5 yr; 100	5 yr; 72	NR	NR
*Studies of genomic classification systems*						
Fernández-Abad et al.^15^	10.4	3.4	NR	NR	NR	NR
McGrath et al.^21^	9.3	5.1	5 yr; 86%	5 yr; 70%	5 yr; 86	5 yr; 70%
Nakagomi et al.^22^	9.9	7.5	NR	NR	NR	NR
Rassy et al.^25^	NR	NR	9.6 yr	6.1 yrs	NR	NR

*IBTR* ipsilateral breast tumour recurrence, *NP* new primary, *NR* not reported, *MD* median, *LR* true local recurrence, *Yr* year.

^a^Survival reported from primary breast cancer.

^b^Survival baseline unclear.

OS data were reported for 11 classification systems: but the survival endpoints varied, with studies reporting 5-, 8- and 10-year OS, or median OS from diagnosis^8,17,19–21,23–25,27,28,30^. OS was generally reported from IBTR diagnosis, with the exception of Komoike et al^8^ who reported survival from primary operation and Sarsenov et al. whose survival baseline was not reported^27^. Where reported, OS from IBTR was consistently higher for NP compared to LR tumours. The 5-year OS ranged from 86 to 100% for NP tumours and 55–76% for LR tumours, while the 10-year OS ranged from 55 to 92% for NP and 44–71% for LR tumours.

10-year DSS data were reported for nine classification systems^17,18,24,28^, and 5-year DSS by one further system^21^. Similar to OS, DSS was generally reported from IBTR diagnosis, with the exception of Jobsen et al. and Smith et al. whose survival baseline was not reported^18,28^. Consistent with OS, DSS where reported, was consistently higher for NP compared to LR tumours. 10-year DSS ranged from 61–90% for NP tumours and 49–68% for LR tumours.

### Management of IBTR

Ten studies reported on the management of IBTR stratified by NP/LR status^6,7,17,19–21,23–25,30^. Across these studies, mastectomy was the most frequently performed surgical intervention for both groups, with IQRs of 73–87% for NP tumours and 62–79% for LR tumours. The use of BCS for IBTR was comparable between groups, with IQRs of 9–28% for NP tumours and 14–35% for LR tumours.

## Discussion

This paper provides a systematic review of the current methods used to classify IBTR as either NP or LR. There were 25 classification systems described across 19 studies, 21 based on clinicopathological criteria and 4 on genomic approaches. A key limitation shared across these systems is the lack of robust validation for their accuracy in distinguishing between NP and LR, compounded by marked heterogeneity across studies, which were often ambiguous or lacking in sufficient detail. These issues substantially limit the reliability and clinical utility of the proposed classification systems.

Despite variations in the definitions and interpretation of individual criteria, there was some consensus regarding which clinicopathological features to include in the classification systems, with concordant location and similar histological sub-type between the PBC and IBTR utilised in 18 (72%) and 16 (68%) of the 25 systems reviewed respectively. However, the definition of concordant location varied and ranged from PBC and IBTR occurring within the same breast quadrant, to IBTR within 3 cm of the primary tumour, or IBTR located within or adjacent to the radiotherapy boost area.

In the largest study included in this review, Wang et al. identified 5413 IBTRs from the Surveillance, Epidemiology and End Results (SEER) database and classified them using the clinicopathological criteria of concordant tumour location and histology, with ER/progesterone receptor (PR) status considered when tumour location was ambiguous^7^. They found that a longer time to IBTR was associated with improved breast cancer specific survival, with LRs tending to recur earlier than NPs. Although this study benefits from a large population, the implications of it’s findings are difficult to interpret given that the study arose from SEER registry records, which have already applied multiple primary rules that attempt to distinguish true recurrence from new primaries, and record the latter only^31,32^.

Shorter time interval to IBTR has been consistently associated with shorter DMFS and OS^33,34^. One proposed explanation is that early recurrences reflect a biologically distinct process from late recurrences, with LRs explaining the majority of the former and NPs most of the latter. This distinction positions time to IBTR as a potentially important surrogate marker for classification. Indeed, it was the most frequently used measure of construct validity across studies in this review, employed in 17 of 19 studies. With the exception of the findings by Laird et al., where time to recurrence results lacked significance, all studies reported a longer interval to recurrence for tumours classified as NP^20^.

Jobsen et al.’s study population represented an outlier, exhibiting substantially longer times to IBTR compared to other cohorts^18^. As their analysis included six classification systems, this disproportionately influenced the overall IQR for time to recurrence across the review, reducing the observed differences between NP and LR.

The distinction in time to IBTR between NP and LR was most pronounced in studies using genomic classification systems, suggesting that these may offer superior construct validity relative to clinicopathological approaches^15,21,22,25^. In the two studies where more than one classification system to the same cohort^6,18^ there was broad agreement across the systems on time to IBTR within the individual study populations.

Among the four studies reporting CBC rates, three found a significantly higher rate in patients with NP tumours, while Panet-Raymond et al. observed similar rates^6,17,23,24^, possibly reflecting a genetic predisposition in patients with NP tumours to develop additional malignancies. Some studies compared the overall rate of CBC with IBTR rates, operating under the expectation that CBC rates may approximate NP rates, whereas IBTR rates would be higher overall due to the additional contribution of LR events. Kraus et al. reported that within the first 5 years from PBC, the likelihood of developing a CBC was significantly greater than that of IBTR, after which the incidence of IBTR and CBC became comparable^19^. This pattern suggests that the early difference is largely attributable to true recurrences, further supporting the use of time to IBTR as a measure for assessing the validity of classification systems.

Clinicopathological classification systems utilise routinely available information from both the PBC and IBTR, thereby facilitating their application within MDT settings and minimising the time and financial costs associated with molecular or genetic profiling. Despite these practical advantages, such systems are subject to inherent limitations: the risk of misclassification undermines their utility for guiding clinical decision making, using NP and LR rates as performance indicators, or evaluating treatment efficacy in research. Misclassification may arise from two principal sources: intrinsic inaccuracies within the classification criteria or from inconsistencies in pathological reporting.

Systems that classify recurrence based on concordance of histological subtype, or receptor status, may overestimate NP, as they fail to account for phenotypic conversion such as gain or loss of HER2 and/or ER expression following treatment of the PBC which could affect one quarter of patients^35,36^. In their study of 35 paired PBC and IBTR specimens, Fernández-Abad et al. used immunohistochemistry, fluorescence in situ hybridisation (FISH) and parallel sequencing to assess clonality. They observed phenotypic conversion in 17% of LRs, all of which shared at least one pathogenic mutation or amplification in a known breast cancer driver gene with the PBC but would have been misclassified as NP by clinicopathological criteria alone^15^. Conversely, 71% of tumours classified as NP retained the same HER2 and ER status as the PBC, increasing their likelihood of being classified as a LR by clinicopathological classification systems. Given that the vast majority of PBC’s are of ductal/no special type and ER-positive, HER2-negative, these clinicopathological systems alone have limited sensitivity for distinguishing NP from LR^37^. While shared pathogenic mutations offer a more objective means of distinction, this approach is not definitive, with the accuracy of genomic systems assessing clonality uncertain. Incorporating additional immunohistochemical markers into existing clinicopathological classification systems, such as basal cytokeratins, neuroendocrine markers, androgen receptor, p53 and S100, could complement assessment of histological subtype and grade, improving the accuracy of IBTR classification; however, none of the reviewed studies employed this approach^38^.

West et al. used the classification criteria described by Panet-Raymond et al. to validate their hypothesis that differing TIL fractions within NP and LR could be used as a biomarker to distinguish between the two^24,29^. They observed reduced TIL responses in NPs compared to LRs, concluding that the approach shows promise. Unfortunately, since West’s initial 2011 study, there has been little research to further develop the use of TILs in distinguishing IBTRs.

The subjectivity of pathological assessment further contributes to classification uncertainty. Despite the adoption of standardised reporting guidelines, inter-observer variability persists^39^. The level of agreement between pathologists on histological subtype classification varies by subtype, with lesions such as pure mucinous and lobular cancers showing greater reproducibility than others^40^. Similarly, there is greater agreement for both grade 1 and grade 3 cancers than for grade 2 carcinomas^40^.

Inaccurate assessment and measurement of surgical margins represents an additional potential source of error, given that excised breast specimens cannot be evaluated in three dimensions. Notably, only 20% of the classification systems reviewed incorporated surgical margin status into their criteria, despite its potential influence on recurrence interpretation. Moreover, some studies excluded patients with positive margins from their cohorts entirely ^24^. The increasing complexity of oncoplastic surgical techniques further complicates spatial assessment, making it more challenging to determine whether an IBTR arises within the same anatomical location as the PBC.

Whilst, in the older literature, considerable variation in hormone receptor status was described, largely due to methodological differences and fixation^41^, the publication of guidelines for hormone receptor and HER2 testing have seemingly resolved much of these discrepancies, for example when samples are re-tested in difference institutions^42^. Nevertheless, misclassification due to false positive or false negative assessment of receptor status remains a consideration. Combined, these factors undermine the reliability of basing this classification and resultant clinical decision making on clinicopathological features alone.

The presence of a non-invasive component in IBTR is a lesser used criterion in clinicopathological classification systems, incorporated in six of the 21 systems reviewed^6,17,20,23,26,28^. Five of these systems shared the hypothesis that the presence of DCIS in IBTR supports NP classification, arguing that once breast cancer becomes invasive, it does not revert to a non-invasive form^6,17,20,23,28^. Sakai et al.’s classification system permitted the presence of in situ components in both NP and LR tumours, depending on the location of the IBTR and the margin status of the PBC excision^26^. However, the differences in the mean time to IBTR for patients labelled as LR and NP in the six classification systems incorporating the presence of DCIS were similar to those that did not include this factor, suggesting its added benefit might be limited.

There are also limitations to using DCIS as a primary criterion for IBTR classification. DCIS often coexists with invasive tumours, around 25% of patients diagnosed with DCIS on core biopsy will be found to have invasive ductal carcinoma following surgical resection^43^. Up to 20% of patients with isolated DCIS develop recurrent DCIS or invasive ductal/ no special type carcinoma^44^. This suggests that a LR could present with an in-situ component despite complete surgical resection of the primary tumour and that the presence of DCIS alone cannot reliably distinguish NP from LR. The work of Idvall et al., using data from the Southern Sweden cancer registry, supports the position that most IBTRs are in fact LRs arising from residual DCIS^45^. Their analysis showed higher concordance between IBTR and the primary tumour (in terms of grade and ER/PR status) than between the primary tumour and contralateral tumours, supporting this interpretation. Similarly, genomic studies of both invasive and non-invasive recurrences have shown clonal relatedness to the original DCIS, suggesting that many IBTRs arise from residual DCIS and are better classified as LR^44^. The presence of DCIS in the PBC or IBTR appears to be relevant, but consistent reporting and subgroup analysis of this characteristic is required in future studies to assess its value in distinguishing NP from LR.

McGrath et al compared a genomic classification method, using a polymerase chain reaction (PCR) based allelic imbalance assay in 57 patients treated with BCS, with a clinicopathological system that classified tumours by location, supplemented with histology when ambiguous, to evaluate the construct validity of their genomic approach^21,46^. They found 44% of tumours were classified differently by the two approaches. 36% of the tumours deemed to be LR by clinicopathological criteria were found to be genetically distinct NPs and 55% of clinicopathological NP tumours were found to be clonally related LR by PCR. Although these comparative findings are insightful, the analysis may have favoured the genomic approach, as the clinicopathological system used was limited, relying primarily on location. In this review, only one study used location alone as the differentiating criterion; it is reasonable to assume that systems incorporating additional clinicopathological features may offer greater classification accuracy^19^.

Nakagomi et al. similarly questioned the reliability of location based systems. Their study compared a genomic profiling technique to a combination of two clinicopathological systems included in this review, requiring concordance in location and histology to define a LR^17,19,22^. Overall, they observed discordance between the two methods in nearly one-quarter of cases. They found that all cases classified as NP by clinicopathological criteria but LR by genomics occurred in a different quadrant of the breast from the PBC. Likewise, tumours reclassified by genomics as NPs despite being designated LRs clinicopathologically were also distinguished solely by location. Given that breast quadrants lack true anatomical boundaries and instead represent nominal divisions for clinical description, it follows that recurrences may not be confined to the same quadrant, challenging the validity of location separate from proximity as a sole distinguishing feature.

With genomic classification offering potentially improved accuracy, further research is needed to rigorously test such methods in larger cohorts. Beyond this, a comprehensive cost benefit analysis is crucial to determine the potential clinical impact of such systems. Importantly, the PBC and IBTR could be sequenced concurrently, removing the need for baseline sequencing at the time of initial PBC diagnosis. However, unlike clinicopathological assessments, the turnaround time for sequencing would still limit its utility for immediate post-operative decision-making at the MDT meeting.

This review has several strengths. It adopts a systematic approach, adhering to Preferred Reporting Items for Systematic Reviews and Meta-Analyses (PRISMA) guidelines, to address a clinically important and currently unresolved question: the accuracy and feasibility of existing methods for classifying IBTR as either NP or LR, and the clinical implications of such a distinction. A comprehensive search across four electronic databases, supplemented by reference list screening of three narrative reviews, ensured the inclusion of studies from a broad range of countries, reducing the risk of omitting relevant evidence.

However, the review is limited by the retrospective nature of all included studies, which do not adequately account for the potential influence of PBC treatment pathways, as well as patient and tumour characteristics, on IBTR classification. Inconsistencies in study design and outcome reporting further limit the comparability of findings. Moreover, the lack of robust methodology to validate proposed classification systems constrains this review’s ability to offer evidence based recommendations for best practice.

The findings of this review underscore the importance of reliable IBTR classification in guiding appropriate surgical decision making. The ability to reliably distinguish IBTR as either NP or LR has significant clinical relevance, particularly when prior treatment history, such as radiotherapy, constrains future therapeutic options^9^. Robust classification could ultimately inform the development of guidelines for second breast-conserving surgery in the context of IBTR. There are also important implications for systemic therapy, if an IBTR represents a LR following prior chemotherapy, re-challenging with the same regimen would be inappropriate; however, if it represents a NP, repeat treatment may be justified within the limits of cumulative toxicity

Although recent data suggest a decline in IBTR rates, potentially lower among those treated with BCS rather than mastectomy, the rising incidence of breast cancer means that IBTR continues to represent a substantial burden in current practice^47–49^. This is particularly pertinent for specific subgroups, such as young women with breast cancer who are at heightened risk of IBTR possibly due to their tendency for aggressive tumour biology, and long potential for survivorship due to a lower risk of death from other conditions^50–52^.

Beyond immediate treatment decisions, establishing a robust system to classify IBTRs has wider implications for data capture, service evaluation and quality improvement. Standardised criteria would enhance the accuracy of cancer registry submissions, enable cumulative evidence generation across institutions, and facilitate meaningful outcome monitoring. In this context, LR rates could serve as a valid performance indicator for treatment quality, given that LRs may reflect some factors within surgical control, whereas NPs are less likely to be influenced by the initial surgical intervention. Such a metric could support efforts to standardise and monitor surgical care at a national level.

To conclude, this review examines the accuracy and feasibility of applying existing classification systems for distinguishing NP from LR breast tumours—a clinically important distinction required to improve outcome recording, and the optimisation of treatment. Accurately identifying these groups could allow for targeted, intensive therapies to improve the poorer outcomes associated with LR tumours, while also supporting the development of evidence based strategies for de-escalating treatment in the more favourable NP group. However, the findings of this review highlight that little progress has been made towards this goal in the past two decades. There remains a lack of studies utilising robust methodology to assess the validity of their proposed classification systems, compounded by the absence of a widely accepted construct for what characteristics should differ between the NP and LR groups. Consequently, there is no high quality evidence to suggest that any of the existing systems reliably reflect the true distribution of NP and LR tumours in clinical practice. None of the existing methods can be recommended for widespread adoption.

Further research employing rigorous prospective methodology is essential to validate the inclusion of criteria commonly used in both clinicopathological and genomic classification systems. Among emerging approaches, assessing tumour clonality between PBC and IBTR may represent a potential way forward. However, implementing clonality based systems poses significant challenges for clinicians and healthcare systems. These include the financial costs, increased molecular pathology workload and the logistical complexities if nationwide implementation were to be proposed.

In the meantime, clinicians must continue to make pragmatic, clinicopathology-based decisions, given the limited and variable availability of genomic clonality testing. IBTRs arising immediately adjacent to the previous surgical scar, particularly where PBC resection margins were positive, can be considered with reasonable confidence as a LR and conversely a cancer situated distant from the primary location, with distinct histopathological features as a NP. Most cases, however, are more nuanced. Features that should increase suspicion of NP include a change in histological subtype, a shift in grade by two levels (e.g. grade 1 to grade 3 or vice versa), conversion from negative to strongly positive hormone receptor status, or the development of HER2 positivity, which is less likely to represent a treatment effect.

In summary, while distinguishing NP and LR tumours remains an important clinical goal, substantial methodological, logistical and economic barriers must be addressed before reliable, evidence-based classification can be implemented in routine practice.

## Methods

### Study eligibility

This systematic review was reported in adherence with the PRISMA guidelines^53^ (see Supplementary 4). Studies were eligible for inclusion if they were primary research studies that assessed the validity of one or more classification systems designed to distinguish IBTR as either LR or NP in an adult population previously treated for primary breast cancer (PBC—invasive or non-invasive) with BCS, with or without radiotherapy. Studies including mixed populations of patients treated with either breast-conserving surgery or mastectomy were eligible for inclusion. Genomic studies were included if they analysed matched pairs of specimens from the original tumour and the subsequent IBTR.

Studies were excluded if they reported IBTR as a single undifferentiated group without subclassification; focused exclusively on contralateral or distant recurrence; addressed recurrence in contexts unrelated to this review’s objectives; were not published in English; were published before 2000; or were available only as conference abstracts. Genomic studies were additionally excluded if published prior to 2010 or if they included fewer than 20 matched primary and recurrent tumour pairs.

### Search strategy

The final search strategy was constructed and performed with support from the Royal College of Surgeons of England evidence support team, and combined terms for ipsilateral breast cancer, recurrence and classification (Supplementary 5). The Electronic searches of Medline, Embase, Cochrane Central Register of Controlled Trials and Cochrane Database of Systematic Reviews were conducted in January 2025 via the Ovid Platform. Reference lists of three review articles^54–56^ and the MARECA study protocol^57^ were scanned for additional studies. A single author (SB) scanned titles and abstracts, selecting relevant articles for full text review. Two authors (SB and JMB) independently performed a full text review for final selection against the inclusion and exclusion criteria, with disagreements resolved in discussion with a senior author (DAC). Publications excluded following full text review are detailed in Supplementary 6.

### Data extraction

Two authors (SB and JMB) extracted data on study characteristics, including article details (author, publication year, country), study design, classification system used, methods for assessing classification performance, and outcomes measured following IBTR. For each study, patient and tumour characteristics, as well as key findings for both NP and LR groups, were collected. Where available, data on survival outcomes and treatment details for IBTR were also extracted. Any reference to the implications of misclassification or the influence of age on classification was noted.

### Study quality assessment

A formal assessment of the quality of the studies was not undertaken because the available quality assessment tools were not considered suitable for this type of research. Instead, the strengths and weaknesses of the included studies are described in narrative form.

### Analysis of results

A narrative synthesis approach was adopted to analyse and report the results, due to the high degree of heterogeneity across studies. Variability in study design, classification systems, outcome measures and methods used to assess classification performance precluded the possibility of a meta-analysis.

## Supplementary information

Supplementary Materials

## Data Availability

All data generated or analysed during this study are included in this published article and its supplementary tables.
