# Supplementary material for: Evaluation of methods to classify ipsilateral breast tumour recurrences as local recurrence or new primary tumour
Source: NPJ Breast Cancer. 2026 Feb 13;12:63. doi: 10.1038/s41523-025-00850-8 (PMC13129046; doi:10.1038/s41523-025-00850-8)
Supplement: Supplementary file 1 — Supplementary Materials [file 41523_2025_850_MOESM1_ESM.pdf]

SUPPLEMENTARY MATERIALS

Supplementary 1: Additional characteristics of studies selected for the systematic review (n= 19)

Author (year)	Design	Exclusion criteria	Adjuvant radiotherapy use for PBC	Median follow-up from PBC (years)	Median follow-up from IBTR (years)
Gujral et al. (2011)	Cohort	NR	100% WBRT +/- boost	10.1	NR
Huang et al. (2002)	Cohort	Poor data quality in records	100% WBRT +/- boost	12.4	7.0
**Jobsen et al. (2022)**	Cohort	Synchronous bilateral BC	100% RT	NR	5.9
**Komoike et al. (2005)**	Cohort	Primary systemic therapy, History of BC	NR	NR	NR
Krauss et al. (2004)	Cohort	NR	100% WBRT +/- boost	NR	4.7
Laird et al. (2018)	Cohort	Inflammatory BC, NACT, Missing ER/PR/HER2 status	100% WB/PBRT +/- boost**	NR	5.4
**Nishimura et al. (2005)**	Cohort	NR	41.6% WBRT	6.2	NR
**Panet-Raymond et al. (2011)**	Cohort	Inflammatory BC, unknown nodal status, previous or synchronous contralateral BC, close or positive margins	87% WBRT +/- boost**	11.4*	LR 5.8*, NP 5.3*
Sakai et al. (2015)	Cohort	NACT, Bilateral BC, synchronous DM	32% RT**	NR	6.3
**Sarsenov et al. (2016)**	Cohort	BCS without RT, NACT, synchronous contralateral recurrence or DM	100% WBRT +/- boost	5.8	NR
**Smith et al. (2000)**	Cohort	NR	100% WBRT +/- boost	14.2*	10.4*
**Wang et al. (2021)**	Cohort	Synchronous bilateral BC or DM	100% RT	13	5.3
**West et al. (2011)**	Cohort	NR	87.5% RT**	NR	NR
**Yi et al. (2011)**	Cohort	NR	84.1% RT**	12.2*	NR
**Yoshida et al. (2010)**	Cohort	NACT, NART, synchronous bilateral BC	83.6% WBRT	5.4	NR

**Fernández-Abad et al. (2025)**	Cohort	isolated LN recurrence	NR	NR	NR
**McGrath et al. (2010)**	Cohort	Tissue blocks unsuitable for genetic analysis	100% WB/PBRT**	10.7	2.1
**Nakagomi et al. (2022)**	Cohort	NR	98% WBRT	7.7	NR
**Rassy et al. (2023)**	Cohort	Tissue blocks unsuitable for genetic analysis	72.9% RT**	NR	4.8

Abbreviations: BC: Breast Cancer; DM: Distant Metastases; ER: Oestrogen Receptor; HER2: Human Epidermal Growth Factor Receptor 2; IBTR: Ipsilateral Breast Tumour Recurrence; NACT: Neoadjuvant Chemotherapy; NART: Neoadjuvant Radiotherapy; NP: New Primary; NR: Not Reported; MD: Median; MC: Multicentre; PBC: Primary Breast Cancer; PBRT: Partial Breast Radiotherapy; PR: Progesterone Receptor; RT: Radiotherapy; SC: Single Centre; LR: True Local Recurrence; WBRT: Whole Breast Radiotherapy. *Mean rather than median. **RT reported as a percentage of the IBTR cohort only, not of the initial PBC population.

Supplementary 2: Distribution of NP and LR tumours across each classification system

Author	Patient Total	Total IBTR no. (IBTR rate, %)	IBTR analysed	NP (% of IBTR)	LR (% of IBTR)	Unclassified (% of IBTR)
Studies of clinicopathological classification systems						
**Gujral et al. (2011)**	1410	150 (11)	150	27 (18)	118 (79)	5 (3)
**Huang et al. (2002)**	1339	139 (10)	126	48 (38)	78 (62)	0 (0)
**Jobsen et al. (2022) - Huang**	4359	234 (5)	234	115 (49)	118 (50)	1 (0)
**Jobsen et al. (2022) - Komoike**	4359	234 (5)	234	110 (47)	121 (52)	3 (1)
**Jobsen et al. (2022) - Morphology**	4359	234 (5)	234	106 (45)	114 (49)	14 (6)
**Jobsen et al. (2022) - Panet-Raymond**	4359	234 (5)	234	132 (56)	100 (43)	2 (1)
**Jobsen et al. (2022) - Twente**	4359	234 (5)	234	138 (59)	90 (38)	6 (3)
**Jobsen et al. (2022) - Yi**	4359	234 (5)	234	136 (58)	93 (40)	5 (2)
**Komoike et al. (2005)**	1901	172 (9)	172	26 (15)	135 (78)	11 (6)
**Krauss et al. (2004)**	1448	79 (5)	79	20 (25)	59 (75)	0 (0)
**Laird et al. (2018)**	3932	115 (3)	81	54 (67)	27 (33)	0 (0)
**Nishimura et al. (2005)**	2137	83 (4)	83	42 (51)	41 (49)	0 (0)
**Panet-Raymond et al. (2011)**	6020	289 (5)	289	139 (48)	129 (45)	21 (7)
**Sakai et al. (2015)**	3876	96 (2)	96	55 (57)	41 (43)	0 (0)

**Sarsenov et al. (2016)**	1400	53 (4)	53	20 (38)	33 (62)	0 (0)
**Smith et al. (2000)**	1152	136 (12)	136	70 (51)	60 (44)	6 (4)
**Wang et al. (2021)**	168,427	5413 (3)	5413	2926 (54)	2487 (46)	0 (0)
**West et al. (2011)**	NR	289	24	12 (50)	12 (50)	0 (0)
**Yi et al. - Method 1**	5660	447 (8)	397	196 (49)	201 (51)	0 (0)
**Yi et al - Method 2**	5660	447 (8)	397	212 (53)	185 (47)	0 (0)
**Yoshida et al. (2010)**	2075	60 (3)	60	8 (13)	52 (87)	0 (0)
Studies of genomic classification systems						
**Fernández-Abad et al. (2025)**	NR	85	35	7 (20)	24 (69)	4 (11)
McGrath et al. (2010)	NR	57	57	23 (40)	34 (60)	0 (0)
**Nakagomi et al. (2022)**	1881	52 (3)	22	8 (36)	14 (64)	0 (0)
**Rassy et al. (2023)**	NR	131	96	79 (82)	17 (18)	0 (0)

Abbreviations: IBTR: Ipsilateral Breast Tumour Recurrence; NP: New Primary; NR: Not Reported; LR: True Local Recurrence

Supplementary 3: Key patient and tumour characteristics by NP & LR status

**Author**	**Median age at primary diagnosis (years)**		**Primary tumour n (%) ER Positive**	
	**NP**	**LR**	**NP**	**LR**
Studies of clinicopathological classification systems				
Gujral et al. (2011)	53	49	NR	NR
Huang et al. (2002)	MN 51.2	MN 50.8	37 (77)	41 (53)
**Jobsen et al. (2022) - Huang**	70	66	85 (74)	100 (85)
**Jobsen et al. (2022) - Komoike**	70	66	80 (73)	103 (85))

**Jobsen et al. (2022) - Morphology**	51	56	75 (71)	98 (87)
**Jobsen et al. (2022) - Panet-Raymond**	69	65.5	94 (71)	91 (91)
**Jobsen et al. (2022) - Twente**	54	54	97 (70)	84 (94)
**Jobsen et al. (2022) - Yi**	67	67	97 (71)	87 (95)
**Komoike et al. (2005)**	47.1	44.8	NR	NR
**Krauss et al. (2004)**	NR	NR	NR	NR
**Laird et al. (2018)**	55.6	60.1	NR	NR
**Nishimura et al. (2005)**	NR	NR	NR	NR
**Panet-Raymond et al. (2011)**	52	52	87 (63)	79 (61)
**Sakai et al. (2015)**	MN 47.9	MN 45.2	NR	NR
**Sarsenov et al. (2016)**	46.4	45.3	11 (55)	16 (49)
**Smith et al. (2000)**	MN 48.9	MN 54.5	31 (66)	29 (69)
**Wang et al. (2021)**	NR	NR	2232 (76)	1870 (75)
**West et al. (2011)**	52	58	8 (67)	9 (75)
**Yi et al. - Method 1**	47.6	47.4	99 (64)	86 (57)
**Yi et al - Method 2**	47.5	47.5	102 (60)	83 (61)
**Yoshida et al. (2010)**	NR	NR	8 (100)	35 (67)
Studies of genomic classification systems				
**Fernández-Abad et al. (2025)**	NR	NR	NR	NR
**McGrath et al. (2010)**	MN 53.7	MN 56.3	16 (70)	23 (68)
**Nakagomi et al. (2022)**	43	49	8(100)	11(79)
**Rassy et al. (2023)**	54	61	70 (93)	14 (100)

Abbreviations: ER: Oestrogen Receptor; IBTR: Ipsilateral Breast Tumour Recurrence; NP: New Primary; NR: Not Reported; MN: Mean; LR: True Local Recurrence

Supplementary 4: Prisma checklist

Section and Topic	Item #	Checklist item	Location where item is reported
**TITLE**			
Title	1	Identify the report as a systematic review.	Page 1
**ABSTRACT**			
Abstract	2	See the PRISMA 2020 for Abstracts checklist.	Page 2
**INTRODUCTION**			
Rationale	3	Describe the rationale for the review in the context of existing knowledge.	Page 3
Objectives	4	Provide an explicit statement of the objective(s) or question(s) the review addresses.	Page 3-4
**METHODS**			
Eligibility criteria	5	Specify the inclusion and exclusion criteria for the review and how studies were grouped for the syntheses.	Page 4
Information sources	6	Specify all databases, registers, websites, organisations, reference lists and other sources searched or consulted to identify studies. Specify the date when each source was last searched or consulted.	Page 4
Search strategy	7	Present the full search strategies for all databases, registers and websites, including any filters and limits used.	Supplementary 5
Selection process	8	Specify the methods used to decide whether a study met the inclusion criteria of the review, including how many reviewers screened each record and each report retrieved, whether they worked independently, and if applicable, details of automation tools used in the process.	Page 4
Data collection process	9	Specify the methods used to collect data from reports, including how many reviewers collected data from each report, whether they worked independently, any processes for obtaining or confirming data from study investigators, and if applicable, details of automation tools used in the process.	Page 4
Data items	10a	List and define all outcomes for which data were sought. Specify whether all results that were compatible with each outcome domain in each study were sought (e.g. for all measures, time points, analyses), and if not, the methods used to decide which results to collect.	Tables 1-5, Supplementary tables 1 &2
	10b	List and define all other variables for which data were sought (e.g. participant and intervention characteristics, funding sources). Describe any assumptions made about any missing or unclear information.	Tables 1-5, Supplementary tables 1 &2

Study risk of bias assessment	11	Specify the methods used to assess risk of bias in the included studies, including details of the tool(s) used, how many reviewers assessed each study and whether they worked independently, and if applicable, details of automation tools used in the process.	Pages 10-16
Effect measures	12	Specify for each outcome the effect measure(s) (e.g. risk ratio, mean difference) used in the synthesis or presentation of results.	Page 4, tables 1-3, supplementary table 1
Synthesis methods	13a	Describe the processes used to decide which studies were eligible for each synthesis (e.g. tabulating the study intervention characteristics and comparing against the planned groups for each synthesis (item #5)).	Page 4, tables 1-3, supplementary table 1
	13b	Describe any methods required to prepare the data for presentation or synthesis, such as handling of missing summary statistics, or data conversions.	N/A
	13c	Describe any methods used to tabulate or visually display results of individual studies and syntheses.	Tables 1-5, supplementary tables 1 & 2
	13d	Describe any methods used to synthesize results and provide a rationale for the choice(s). If meta-analysis was performed, describe the model(s), method(s) to identify the presence and extent of statistical heterogeneity, and software package(s) used.	Page 4
	13e	Describe any methods used to explore possible causes of heterogeneity among study results (e.g. subgroup analysis, meta-regression).	N/A
	13f	Describe any sensitivity analyses conducted to assess robustness of the synthesized results.	N/A
Reporting bias assessment	14	Describe any methods used to assess risk of bias due to missing results in a synthesis (arising from reporting biases).	N/A
Certainty assessment	15	Describe any methods used to assess certainty (or confidence) in the body of evidence for an outcome.	N/A
**RESULTS**			
Study selection	16a	Describe the results of the search and selection process, from the number of records identified in the search to the number of studies included in the review, ideally using a flow diagram.	Page 5, Figure 1
	16b	Cite studies that might appear to meet the inclusion criteria, but which were excluded, and explain why they were excluded.	Supplementary 6
Study characteristics	17	Cite each included study and present its characteristics.	Pages 6-7, Table 1
Risk of bias in studies	18	Present assessments of risk of bias for each included study.	N/A

Results of individual studies	19	For all outcomes, present, for each study: (a) summary statistics for each group (where appropriate) and (b) an effect estimate and its precision (e.g. confidence/credible interval), ideally using structured tables or plots.	Tables 1-5, Supplementary tables 1-2
Results of syntheses	20a	For each synthesis, briefly summarise the characteristics and risk of bias among contributing studies.	Pages 6-10, Table 1 & Supplementary table 1
	20b	Present results of all statistical syntheses conducted. If meta-analysis was done, present for each the summary estimate and its precision (e.g. confidence/credible interval) and measures of statistical heterogeneity. If comparing groups, describe the direction of the effect.	N/A
	20c	Present results of all investigations of possible causes of heterogeneity among study results.	N/A
	20d	Present results of all sensitivity analyses conducted to assess the robustness of the synthesized results.	N/A
Reporting biases	21	Present assessments of risk of bias due to missing results (arising from reporting biases) for each synthesis assessed.	N/A
Certainty of evidence	22	Present assessments of certainty (or confidence) in the body of evidence for each outcome assessed.	N/A
**DISCUSSION**			
Discussion	23a	Provide a general interpretation of the results in the context of other evidence.	Pages 11-15
	23b	Discuss any limitations of the evidence included in the review.	Page 11-15
	23c	Discuss any limitations of the review processes used.	Page 15
	23d	Discuss implications of the results for practice, policy, and future research.	Pages 15-16
**OTHER INFORMATION**			
Registration and protocol	24a	Provide registration information for the review, including register name and registration number, or state that the review was not registered.	Page 17
	24b	Indicate where the review protocol can be accessed, or state that a protocol was not prepared.	Page 17
	24c	Describe and explain any amendments to information provided at registration or in the protocol.	N/A
Support	25	Describe sources of financial or non-financial support for the review, and the role of the funders or sponsors in the review.	Page 18
Competing interests	26	Declare any competing interests of review authors.	Page 18
Availability of data, code and other materials	27	Report which of the following are publicly available and where they can be found: template data collection forms; data extracted from included studies; data used for all analyses; analytic code; any other materials used in the review.	Page 18

Supplementary 5: Search strategies for Ovid Medline, Embase and Cochrane

Database:	Ovid MEDLINE(R) ALL <1946 to Jan 28, 2025>	Results per line:
Date:	29/01/2024	
1	exp breast neoplasms/	363733
2	((breast* or ductal* or mammar* or Phyllodes) adj2 (cancer* or carcinoma* or neoplasm* or tumo?r* or malign* or metast* or sarcoma*)).ti,ab.	433291
3	1 or 2	510636
4	Ipsilateral.ti,ab,kw,kf.	71163
5	3 and 4	3857
6	Neoplasm Recurrence, Local/	152701
7	Neoplasms, Second Primary/	17677
8	Neoplasm Metastasis/	116588
9	Neoplasm Staging/	199658
10	(recurren* or clones seed* or metatas* or relapse* or metachronous or relapse*).ti,ab,kw,kf.	922656
11	((new or multiple or second) adj2 (tumo?r or cancer* or neoplasm* or reccur* or carcinoma* or malignan*)).ti,ab,kw,kf.	65165
12	6 or 7 or 8 or 9 or 10 or 11	1256272
13	((distinguish* or classif* or misclassif* or determine* or test* or definite* or suspected or characterise*) adj8 (new or primary or second* or true or de nuovo or NP or local recurrence* or LR or IBTR)).ti,ab,kw,kf.	321861
14	3 and 4 and 12 and 13	138
15	limit 14 to yr="2000 - 2025"	121
16	limit 15 to english language	119

Database:	Embase <1974 to 2025 Week 04>	Results per line:
Date:	29/01/2025	
1	exp breast tumor/	713957
2	((breast* or ductal* or mammar* or Phyllodes) adj2 (cancer* or carcinoma* or neoplasm* or tumo?r* or malign* or metast* or sarcoma*)).ti,ab.	614318
3	1 or 2	791935
4	Ipsilateral.ti,ab,kw,kf.	94080
5	3 and 4	6943
6	exp cancer recurrence/	302708
7	tumor recurrence/	73847
8	second cancer/	16307
9	breast cancer recurrence/	1601
10	cancer staging/	484455
11	(recurren* or clones seed* or metatas* or relapse* or metachronous or relapse*).ti,ab,kw,kf.	1424909
12	((new or multiple or second) adj2 (tumo?r or cancer* or neoplasm* or reccur* or carcinoma* or malignan*)).ti,ab,kw,kf.	99187
13	6 or 7 or 8 or 9 or 10 or 11 or 12	1936724
14	((distinguish* or classif* or misclassif* or determine* or test* or definite* or suspected or characterise*) adj8 (new or primary or second* or true or de nuovo or NP or local recurrence* or LR or IBTR)).ti,ab,kw,kf.	446198
15	3 and 4 and 13 and 14	274
16	limit 15 to yr="2000 - 2025"	256

17	limit 16 to english language	253

Database:	Cochrane Central Register of Controlled Trials (CENTRAL) and Cochrane Database of Systematic Reviews (CDSR)	Results per line:
Date:	03/02/2025	
#1	MeSH descriptor: [Breast Neoplasms] explode all trees	20844
#2	((breast* or ductal* or mammar* or Phyllodes) near/2 (cancer* or carcinoma* or neoplasm* or tumo?r* or malign* or metast* or sarcoma*)):ti,ab	44809
#3	#1 or #2	46940
#4	Ipsilateral:ti,ab,kw	4014
#5	#3 and #4	757
#6	MeSH descriptor: [Neoplasm Recurrence, Local] this term only	7732
#7	MeSH descriptor: [Neoplasms, Second Primary] this term only	627
#8	MeSH descriptor: [Neoplasm Metastasis] this term only	4709
#9	MeSH descriptor: [Neoplasm Staging] this term only	10161
#10	(recurren* or clones seed* or metatas* or relapse* or metachronous or relapse*):ti,ab,kw	125200
#11	((new or multiple or second) NEAR/2 (tumo?r or cancer* or neoplasm* or reccur* or carcinoma* or malignan*)):ti,ab,kw	4781
#12	#6 or #7 or #8 or #9 or #10 or #11	138110
#13	((distinguish* or classif* or misclassif* or determine* or test* or definite* or suspected or characterise*) NEAR/8 (new or primary or second* or true or de nuovo or NP or local recurrence* or LR or IBTR)):ti,ab,kw	87790
#14	#3 and #4 and #12 and #13	81
#15	#14 with Publication Year from 2000 to 2025, in Trials	72

Supplementary 6 - Publications excluded following full text review

Abd-Alla HM, Lotayef MM, Abou Bakr A, Moneer MM. Ipsilateral in-breast tumor relapse after breast conservation therapy: true recurrence versus new primary tumor. J Egypt Natl Canc Inst. 2006;18(3):183-90.

Biermann J, Parris TZ, Nemes S, Danielsson A, Engqvist H, Werner Rönnerman E, et al. Clonal relatedness in tumour pairs of breast cancer patients. Breast Cancer Res. 2018;20(1):96.

Freedman GM, Anderson PR, Hanlon AL, Eisenberg DF, Nicolaou N. Pattern of local recurrence after conservative surgery and whole-breast irradiation. Int J Radiat Oncol Biol Phys. 2005;61(5):1328-36.

Huang KT, Mikeska T, Li J, Takano EA, Millar EK, Graham PH, et al. Assessment of DNA methylation profiling and copy number variation as indications of clonal relationship in ipsilateral and contralateral breast cancers to distinguish recurrent breast cancer from a second primary tumour. BMC Cancer. 2015;15:669.

Hwang SH, Lee JW, Son BH, Jeong J, Ahn SH, Ahn S-G, et al. 252 Comparison of True Recurrence Versus New Primary: an Analysis of Ipsilateral Breast Tumor Recurrences After Breast-Conserving Therapy. Eur J Cancer. 2012;48:S116.

Kader T, Zethoven M, Mahale S, Saunders H, Tjoeka L, Lehmann R, et al. Predictive biomarkers of breast ductal carcinoma in situ may underestimate the risk of recurrence due to de novo ipsilateral breast carcinoma development. bioRxiv. 2024:2024.05.19.594731.

Luo S, Su X, DeSantis SM, Huang X, Yi M, Hunt KK. Joint model for a diagnostic test without a gold standard in the presence of a dependent terminal event. Stat Med. 2014;33(15):2554-66.

Luo S, Su X, Yi M, Hunt KK. Simultaneous inference of a misclassified outcome and competing risks failure time data. J Appl Stat. 2015;42(5):1080-90.

Luo S, Yi M, Huang X, Hunt KK. A Bayesian model for misclassified binary outcomes and correlated survival data with applications to breast cancer. Stat Med. 2013;32(13):2320-34.

Van Alsten SC, Zipple I, Calhoun BC, Troester MA. Misclassification of second primary and recurrent breast cancer in the surveillance epidemiology and end results registry. Cancer Causes Control. 2025;36(4):421-32.
